# Epigenetic Regulation of microRNAs in Cancer: Shortening the Distance from Bench to Bedside

**DOI:** 10.3390/ijms22147350

**Published:** 2021-07-08

**Authors:** María J. Pajares, Ester Alemany-Cosme, Saioa Goñi, Eva Bandres, Cora Palanca-Ballester, Juan Sandoval

**Affiliations:** 1 Biochemistry Area, Department of Health Sciences, Public University of Navarre, 31008 Pamplona, Spain; mjose.pajares@unavarra.es (M.J.P.); saioa.goni@unavarra.es (S.G.); 2 IDISNA Navarra’s Health Research Institute, 31008 Pamplona, Spain; evamaria.bandres@unavarra.es; 3 Biomarkers and Precision Medicine Unit, Health Research Institute la Fe, 460026 Valencia, Spain; ester_alemany@iislafe.es (E.A.-C.); cora_palanca@iislafe.es (C.P.-B.); 4 Immunology Unit, Department of Hematology, Complejo Hospitalario de Navarra, 31008 Pamplona, Spain; 5 Epigenomics Core Facility, Health Research Institute la Fe, 46026 Valencia, Spain

**Keywords:** epigenetics, DNA methylation, microRNAs, cancer, biomarkers, clinical applications

## Abstract

Cancer is a complex disease involving alterations of multiple processes, with both genetic and epigenetic features contributing as core factors to the disease. In recent years, it has become evident that non-coding RNAs (ncRNAs), an epigenetic factor, play a key role in the initiation and progression of cancer. MicroRNAs, the most studied non-coding RNAs subtype, are key controllers in a myriad of cellular processes, including proliferation, differentiation, and apoptosis. Furthermore, the expression of miRNAs is controlled, concomitantly, by other epigenetic factors, such as DNA methylation and histone modifications, resulting in aberrant patterns of expression upon the occurrence of cancer. In this sense, aberrant miRNA landscape evaluation has emerged as a promising strategy for cancer management. In this review, we have focused on the regulation (biogenesis, processing, and dysregulation) of miRNAs and their role as modulators of the epigenetic machinery. We have also highlighted their potential clinical value, such as validated diagnostic and prognostic biomarkers, and their relevant role as chromatin modifiers in cancer therapy.

## 1. Introduction

MicroRNAs (miRNAs) are 18–25-nucleotide-long, non-coding RNAs with critical roles in a variety of biological processes, such as proliferation, differentiation, or immune response. They exert their function through the regulation of gene expression, mostly at the post-transcriptional level [1,2,3,4]. Most miRNA sequences are located within introns of coding genes or in intron and exons of non-coding RNAs [5]. miRNAs regulation occurs mainly due to genetic or epigenetic mechanisms. Epigenetic mechanisms include DNA methylation, post-translational modification of histones, and RNA modification. In addition, a group of miRNAs, called epi-miRNAs, can modulate the expression of DNA methyltransferases (DNMTs), histone deacetylases (HDACs), or histone methyltransferases (HMTs) [6], affecting the expression of both coding and non-coding genes and consequently having a clear impact on the global epigenome. Furthermore, miRNAs can interact with complementary sequences in gene promoters, representing a platform for the assembly of specific protein complexes that regulate gene expression through changes in the chromatin structure [7].

Interestingly, aberrant expression of miRNAs is associated with different diseases, especially cancer. Besides, there is a growing list of reported miRNAs with an oncogenic function (referred to as “oncomiRs”) as well as miRNAs with a tumoral suppressing function (namely “oncosuppressor miRs”) in several neoplastic malignancies. Importantly, their expression and function greatly differ depending on the cancer type or even the cancer stage. Alterations in miRNA expression in cancer have been attributed to genomic variations in miRNA genomic loci, modulation of miRNA expression by transcription factors (TFs), and dysregulation of the miRNA biogenesis. However, epigenetic alterations (i.e., DNA methylation and histone modifications) are the major causes of miRNA dysregulation in cancer [8,9]. Further understanding of the dysregulation of miRNAs and their crosstalk with epigenetic mechanisms may enable the development of novel strategies for cancer prevention, diagnosis, and treatment.

## 2. The Biogenesis of miRNAs

**Figure 1:**
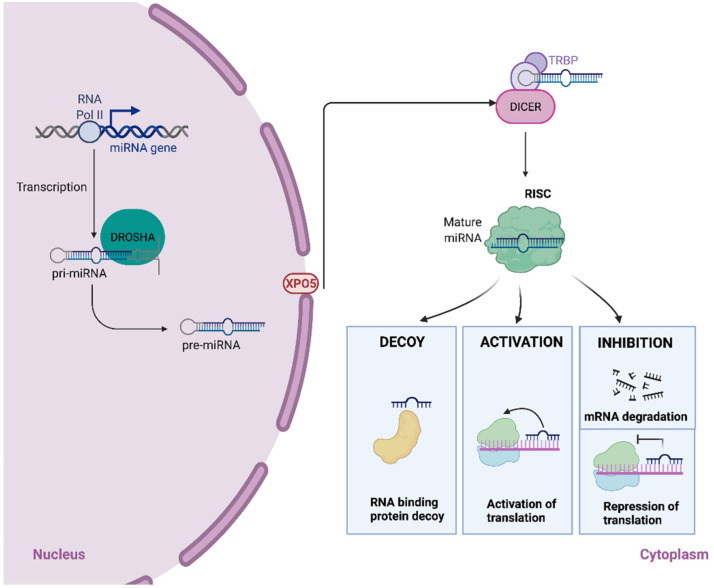
The biogenesis of microRNAs. MicroRNA genes are generally transcribed by RNA Polymerase II in the nucleus to form large pri-miRNA transcripts, which are capped and polyadenylated. These pri-miRNA transcripts are processed by the RNase III enzyme Drosha to release the ~70-nucleotide pre-miRNA precursor product. Exportin 5 (XPO5) transports the pre-miRNA into the cytoplasm. Subsequently, another RNase III enzyme, Dicer, processes the pre-miRNA to generate a transient ~22-nucleotide miRNA: miRNA* duplex. This duplex is then loaded into the miRNA-associated multiprotein RNA-induced silencing complex (mi-RISC). The mature miRNA then binds to complementary sites in the target mRNA to induce an RNA-binding protein decoy, activation of translation, or inhibition of translation by mRNA degradation or translation repression. Created with Biorender.com.

miRNA genes are transcribed by RNA polymerase II to produce long-capped RNA molecules called primary miRNAs (pri-miRNAs) [10]. Pri-miRNAs are cleaved into 60–100 nucleotide-long hairpin precursors, known as pre-miRNAs, by a multiprotein complex that consists of Drosha, a double-stranded RNA specific ribonuclease III, and its cofactor DGCRB8 (DiGeorge syndrome critical region gene 8) [11,12,13]. Subsequently, the exportin 5 transporter (XPO5) translocates the pre-miRNA from the nucleus to the cytoplasm via a RAN-GTP-dependent mechanism [14,15]. The pre-miRNA is processed in the cytoplasm by DICER, a RNAse III endonuclease, into an 18–25 nucleotide-long double-stranded RNA [16]. Finally, the duplex is loaded into the RNA-induced silencing complex (RISC), where one strand is degraded [17]. The RISC complex guides the mature miRNA to its mRNA target, provoking its degradation or repression and consequently a reduction in the protein levels. miRNAs with high-grade complementarity to the target mRNA induce cleavage and degradation, whereas translational repression is observed when miRNAs bind imperfectly to their mRNA target [18,19] (Figure 1).

Activation of translation instead of repression has also been described as a function that can be exerted by some miRNAs [20]. Most miRNAs interact with 3′-untranslated regions (3′-UTR) of mRNAs, but binding to 5′-UTR or coding regions of target mRNAs also has been demonstrated [3,21]. Besides, some miRNAs, such as miR-10, may bind both the 3′-UTR and 5′-UTR of mRNAs, exerting different functions depending on the site of interaction. Thus, miR-10a can repress translation after interacting with the 3′-UTR of a specific mRNA or stimulate translation via binding to the 5′-UTR of a different mRNA [22]. On the other hand, miRNAs can regulate mRNA metabolism by acting as molecular decoys for RNA-binding proteins [23]. Finally, miRNAs could be processed by alternative routes, called non-canonical pathways, bypassing one or more steps of those described above. The main non-canonical miRNA biogenesis pathways are Drosha/DGCR8-independent and Dicer-independent pathways. An example of the former is the so-called mirtron pathway, where an intronic sequence of a particular mRNA could function as pre-miRNA. This intronic sequence is processed by the spliceosome, exported to the cytoplasm by XPO5 to continue with the canonical pathway via DICER to form a mature miRNA [24]. Other miRNA biogenesis pathways have been shown to be Drosha dependent, but either Drosha’s binding partner DGCR8 or Dicer independent [25,26].

## 3. Epigenetic Regulation of miRNAs Expression

Epigenetics refer to inheritable features that are related to alterations or changes outside the DNA nucleotide sequence, giving rise to changes in gene expression patterns. As other coding and non-coding genes, miRNAs have been described to be regulated by epigenetic mechanisms such as DNA methylation, histone, and RNA modifications. Besides, TFs have been shown to facilitate the recruitment of epigenetic regulators to gene promoters, contributing to epigenetic control of gene expression in different scenarios. The work of Ozsolak and collaborators in 2008, identifying the miRNA promoter structures, was important to establish the miRNA expression regulatory mechanisms [5]. In fact, high-throughput analysis of miRNA promoter structures by nucleosome mapping, and H3K4me3 and H3K9/14ac ChIP-Chip screening, confirmed the similarity of the RNAPII-transcribed miRNA promoters and mRNA-encoding promoters regarding the CpG island, TATA elements, TFIIB recognition elements (BRE), initiator (Inr), and other elements [5]. Notably, the nucleosome occupancy information surrounding the miRNA transcription start site (TSS) was also used for the discovery of TF-mediated regulation of miRNAs [5].

### 3.1. DNA Methylation

DNA methylation consists of the covalent addition of a methyl group in cytosine nucleotides (5-methylcytosine, 5-mC), usually within CpG dinucleotides that are concentrated in CpG islands. Around 60% of these CpG islands are located in gene promoter regions, where DNA methylation causes transcriptional repression, enabling the binding of repressor proteins and preventing the interaction between TFs and DNA [27]. 5-mC can also be found in gene bodies and intergenic regions [28], encountering some differences regarding transcription regulation. Similar to promoters, 5-mC accumulation in intergenic regions and repetitive elements is associated with genomic integrity. Remarkably, many CpG islands have been found outside TSS, indicative of unannotated transcripts or enhancer elements [29]. Regarding gene bodies, high levels of 5-mC have been found in highly expressed genes. This apparent paradox has been related to the protection of the gene body from spurious RNA polymerase II entry and cryptic transcription initiation, leading to the fidelity of gene transcription initiation [30]. DNA methyltransferase family enzymes are responsible for the covalent addition of methyl groups, being specific for each substrate. For instance, DNMT1 acts on hemimethylated DNA and maintains the methylation of the newly synthesized strand in DNA replication. DNMT3A and DNMT3B are responsible for de novo methylation of unmethylated DNA. Significantly, DNA hypermethylation in promoter regions is associated with transcriptional repression by different mechanisms, such as the prevention of TF binding, the recruitment of histone deacetylases, and the recruitment of methyl-CpG-binding proteins a with repressive function [31]. In contrast, DNA demethylation is mediated by the Tet methylcytosine dioxygenase (TET) family of enzymes, regardless of DNA replication [32].

Importantly, the first evidence suggesting that miRNAs might be regulated by DNA methylation was published in 2006 [33]. Saito and collaborators treated bladder cancer cells with the demethylating agent 5-Aza-2′-deoxycytidine (5-aza-dC), leading to the upregulation of 17 out of the 313 human miRNAs characterized by a miRNA microarray [33]. Notably, one of these miRNAs, miR-127, was embedded in a CpG island and was significantly upregulated upon treatment, proving for the first time that DNA methylation regulated miR-127 expression. Since then, around 50% of the miRNAs have been described to be located in CpG island-rich positions and therefore to be subjected to epigenetic regulation [34]. The expression of neighboring mRNAs was not analyzed in this study, but Ozsolak and colleagues would confirm that there is no expression correlation between intronic miRNAs having distinct promoters, and their host’s mRNAs [5].

DNA methylation changes have been largely observed in different pathologies, particularly in cancer. The latter are characterized by global genomic demethylation, especially mobile genetic elements, and selective hypermethylation of regions exhibiting tumor-suppressor functions [35].

### 3.2. Histone Modifications

Eukaryotic cells have their DNA highly packed into the nucleus as an assembly of DNA and DNA-interacting proteins, mainly histones. Histones are basic proteins that form the octamer structures (H3, H4, H2A, and H2B), which interact with DNA, leading to the formation of nucleosomes. These are no longer believed to be static entities but very dynamic in the regulation of gene expression [36].

Every histone protein owns a characteristic side chain or tail, which is mainly composed of basic lysine and arginine residues. The histone tails experience extensive covalent post-translational modifications that will ultimately lead to changes in chromatin organization and packaging. The main modifications that have been studied so far are acetylation, methylation, and phosphorylation, but there are many others, such as citrullination, ubiquitination, ADP-ribosylation, deamination, formylation, O-GlcNAcylation, propionylation, butyrylation, crotonylation, and proline isomerization [36].

#### 3.2.1. Histone Acetylation

Acetyl groups can be added at lysine residues on histone tails, leading to the neutralization of the basic charge of histones, mostly localized at the enhancers, promoters, and gene bodies [37]. This modification weakens the interaction between the negatively charged DNA and histones, promoting the active transcription of genes. The most frequent acetylated residues are lysine 27 and lysine 9 from the H3 histone. They are usually placed in enhancers and promoters by typical “writers” (histone acetyltransferases), such as the P300/CREB-binding protein (CBP), and removed by “erasers”, such as HDACs/sirtuins [36]. HDAC proteins have been reported to be altered in a wide variety of disorders, including cancer. Overexpression of HDAC leads to the compaction of chromatin and repression of transcription [38], and thus, the use of different HDAC inhibitors (HDACi) (4-sodium phenylbutyrate (PBA), trichostatin A (TSA), or suberoylanilide hydroxamic acid (SAHA) have led to the discovery of HDAC-repressed miRNAs in cancer.

HDACi treatment in cancer cell lines led to the derepression of several miRNAs, which confirmed the role of HDACs in the regulation of miRNAs. For instance, the induction of miR-200a [39] and miR-200c [40] was observed in breast cancer cells upon HDACi treatment, leading to the inhibition of cell proliferation, invasion, and migration [40]. In pancreatic cancer cell lines, HDACi treatment led to the induction of miR-34a expression, causing the inhibition of tumor-progression-related features, such as cell proliferation, cell cycle progression, epithelial to mesenchymal transition (EMT), and invasion [41]. In line with this, the combination of different HDACi was shown to induce the expression of miR-31 in breast cancer cells, resulting in cellular senescence [42]. These and other examples discussed in Section 5 point out the critical role of HDACs in the regulation of miRNA expression and their dysregulation in cancer.

#### 3.2.2. Histone Methylation

The addition of a specific number of methyl groups (-CH_3_) in specific lysine or arginine residues is tightly regulated, having a pivotal role in gene regulation. Lysine residues in histones can be mono-, di-, or tri-methylated and will have a different effect on gene regulation, depending on the position of the post-translational modification.

Regarding lysine residues, H3K4 trimethylation (localized at gene promoter regions) and H3K4 monomethylation (enriched at enhancer and promoter regions), H3K36 trimethylation (mainly distributed within the gene body), and H3K79 dimethylation (localized both in the promoter and along the gene body) modifications are usually gene-activating marks. On the other hand, H3K9 trimethylation and H3K27 monomethylation marks repress gene expression. Importantly, each modification is associated with specific writers (histone methyltransferases) and erasers (histone demethylases), such as the couple SET Domain Containing 1A/D (SETD1A/D) (writers) and Lysine Demethylase 5A/B/C (KDM5A/B/C) (erasers) in the case of H3K4me3; for H3K36me3, SET Domain Containing 2D (SETD2) and Lysine Demethylase 4 (KDM4); and for H3K4me1, Mixed Lineage Leukemia protein-1-5 (MLL1-5) and Lysine Demethylase 1A/B (KDM1A/B). Finally, for H3K9me3, the writer Suppressor Of Variegation 3–9 Homolog 1/2 (SUV39H1/2) and eraser Lysine Demethylase 3/4(KDM3/4), and Enhancer Of Zeste 1/2 Polycomb Repressive Complex 2 Subunit (EZH2/EZH1) and Lysine Demethylase 6 A/B (KDM6A/B) in the case of H3K27me1 [36]. Noteworthy, several histone methylation modifiers have been related to miRNA regulation, such as EZH2 in ovarian cancer, which has been shown to induce the repressive histone mark H3K27me3 in target miRNAs: miR-101-3p, let-7e-5p, miR-26a-5p, miR-98-5p, and miR-141-3p [43]. In lung cancer cells, KDM5B was shown to inhibit the miR-200 family via demethylation of H3K4me3, inducing EMT of cancer cells [44]. KDM5A upregulation was proven to promote cervical cancer progression by repressing miR-424-5p through directly interacting with its promoter region and removing the H3K4 methyl groups [45]. In Section 5, more details regarding histone modification and miRNA regulation in cancer will be discussed.

### 3.3. RNA Modifications

To date, more than 140 RNA modifications have been discovered as adenosine methylation (m6A), cytosine methylation (m5C), ribose methylation (2′-O-Me), and pseudourylation (Ψ). Among them, m6A is one the most prevalent internal modification of mRNA. m6A has been found in around 7000 genes and is catalyzed by a complex of proteins with methyltransferase activity, including methyltransferase-like 3 (METTL3) and methyltransferase-like 14 (METTL14). It has been shown that the presence of m6A might alter gene expression levels, mRNA stability, translation efficiency, and other relevant functions [46]. Importantly, Alarcon and colleagues found that m6A was also localized in non-coding genes, such as the pri-miRNAs, promoting their recognition by DGCR8 and allowing the genesis of the pre-miRNAs. Besides, they demonstrated that downregulation of m6A by depletion of METTL3 led to a reduction in most mature miRNAs [47].

## 4. miRNAs as Epigenetic Regulators

In the previous section, the epigenetic regulation of miRNAs has been addressed. Conversely, many studies have proven that miRNAs themselves might act as epigenetic regulators (epi-miRNAs), which post-transcriptionally target the factors belonging to the epigenetic machinery, such as DNMTs or DNA-demethylases, histone acetylases or HDACs, and histone methyltransferases (EZH2) or demethylases (KDMs). Epi-miRNAs, likewise other miRNAs, target the 3′-UTRs of the mRNAs, inducing their degradation [48]. The latter enables the regulation of DNA methylation, histone acetylation, and histone methylation, with the consequent changes in global gene transcription. Importantly, epi-miRNAs themselves can be epigenetically regulated, conforming to the regulatory circuits that often appear deregulated in several disorders. Many demethylases have been involved in epi-miRNA reciprocal regulatory loops, such as DNMT3A with miR-29a/b in lung cancer (90) and miR-200c in gastric cancer (103). EZH2 has also been frequently involved in epi-miRNA regulatory loops. For instance, the previously mentioned study from Liu and collaborators [43] confirmed the reciprocal regulation of EZH2 and a set of five epi-miRNAs (miR-101-3p, let-7e-5p, miR-26a-5p, miR-98-5p, and miR-141-3p) in ovarian cancer, promoting malignant proliferation by maintaining the high expression of EZH2. Other examples will be further characterized in Section 5.

In addition, deregulated m6A modification is an important hallmark of various diseases, including cancer. Notably, regarding the regulation of RNA modifications by miRNAs, it was proved that miRNAs regulate m6A RNA methylation by modulating the binding of METTL3 to mRNAs [49].

Outside of the post-transcriptional function of many miRNAs, it has been shown that miRNAs might remain retained in the nucleus, regulating gene activation and silencing, via recruitment of other epigenetic factors [50]. In fact, several miRNAs have been reported to activate gene transcription via enrichment of markers for transcriptionally active promoters (H3K4me3) and recruitment of polymerase II (Pol II) [51]. Regarding gene silencing, many other miRNAs have been proven to repress gene transcription by the recruitment of Polycomb proteins and inducing the H3K27me3, which maintains the chromatin in a condensed form [52].

**Figure 2:**
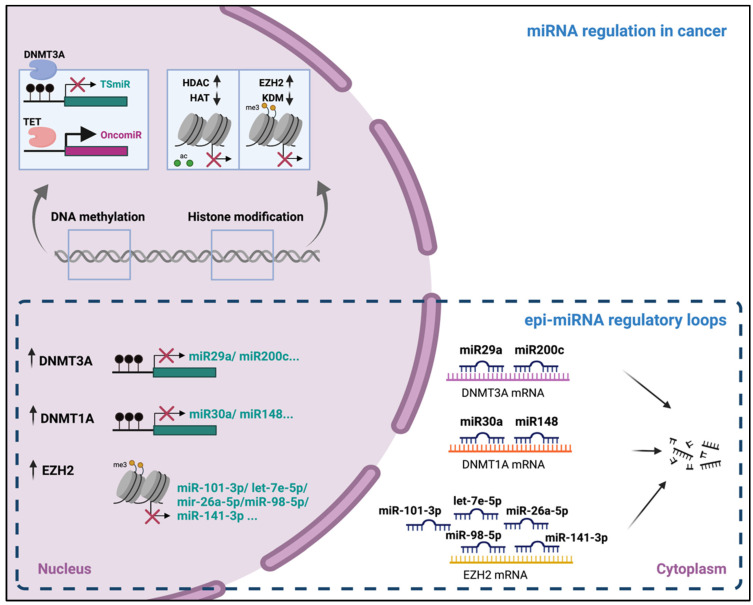
The regulation of microRNAs and epi-miRNAs in cancer. DNA methylation and histone modifications are the major epigenetic mechanisms involved in the regulation of miRNA expression. Many epi-miRNAs have been shown to target epigenetic factors, such as DNMT3A, DNMT1A, and EZH2, and these, in turn, are implicated in the reciprocal regulation of the epi-miRNAs, giving rise to regulatory loops that are often dysregulated in cancer. Created with Biorender.com.

All these mechanisms combine tightly in order to regulate gene expression and, thus, the relevant biological processes. The deregulation of any of the aforementioned mechanisms in cancer might have tremendous effects, disturbing the circuits that control important processes, including cell proliferation or apoptosis (Figure 2).

## 5. Epigenetic Alteration and microRNA Dysregulation in Cancer

### 5.1. The Cancer Epigenome Landscape

Cancer diseases are characterized by extensive epigenetic changes [53]. The first epigenetic abnormality described in human tumors was the loss of DNA methylation back in 1983 [54,55,56]. It is well-established that genome-wide DNA hypomethylation is a frequent feature of human cancers, which can be found in the early stages of carcinogenesis and associated with tumor progression [57]. The overall low levels of genomic DNA methylation is due to the hypomethylation of highly repeated DNA sequences (such as long-interspersed retrotransposable elements (LINEs), short-interspersed retrotransposable elements (SINEs), and long terminal repeats (LTRs)) [58,59,60]. In contrast, the genomic regions associated with hypermethylation are gene regions, mainly localized in promoter-associated CpG islands. In fact, the inactivation or downregulation of tumor-suppressor genes via promoter hypermethylation is commonly observed in most types of human cancers [61,62]. Some examples of silenced genes by CpG-island hypermethylation include the inhibitor of the JAK–STAT pathway SOCS1 in the liver and myeloma tumors or the cell-cycle inhibitor Rb in retinoblastoma tumors [63]. Although the impact of global DNA hypomethylation on cancer is less straightforward than that of the localized hypermethylation, it is also thought to contribute to cancer development by generating chromosomal instability, reactivating transposable elements, or causing the loss of genomic imprinting [53].

On the other hand, an aberrant pattern in the histones’ post-translational modifications in cancer has also been described, leading to the reconfiguration of the entire genome during the tumor process [64,65]. As previously outlined, these modifications in histones are due to alterations in the levels of the regulatory enzymes, such as histone deacetylases (HDAC1, HDAC2) or histone demethylases (lysine-specific demethylase LSD1) [62] and have a preponderant role during EMT [66] and in the regulation of tumoral metastasis [67]. In cancer, deregulation of the histone writers and erasers can lead to the histone hypoacetylation of oncosuppressor miRs or hyperacetylation of oncomiRs [68].

Concerning miRNAs, their dysregulation is also a common feature of human cancers [69,70]. In the past decades, their relevant role in tumor onset, growth, and metastasis has been demonstrated [8]. Generally, the expression of miRNAs is downregulated in tumors compared to their corresponding healthy tissues. This leads to the idea that many miRNAs could be acting as oncosuppressor miRs [9,71]. Nevertheless, overexpression of miRNAs functioning as oncogenes has also been described in human tumors. All in all, it has to be kept in mind that miRNAs can have multiple targets and can function as either tumor suppressors or oncogenes under different circumstances, depending on the tissue or cell type where they exert their function [69,72]. Consequently, to understand the repercussion of miRNA dysregulation, it is crucial to pay attention to cancer-specific miRNA expression patterns. The major causes of miRNA dysregulation in malignant cells are the amplification, deletion, or translocation of the miRNA-encoding genes, abnormal epigenetic modifications, defects in the miRNA biogenesis machinery, or widespread transcriptional repression [73]. It is also noteworthy that a significant number of miRNA genes are located within cancer-associated genomic regions or fragile sites [74].

### 5.2. miRNAs in the Control of Critical Cancer-Related Pathways

Cancer diseases are characterized by the disruption of cellular homeostasis pathways, which ultimately result in uncontrolled cell growth, proliferation, and resistance to apoptosis. miRNAs function as fundamental and versatile gene regulators in cancer since they can target a large number of the pathways that sustain these essential cellular functions [73]. Firstly, miRNAs acting as oncomiRs are typically overexpressed and enable cancerous cells to enter and progress through the cell cycle, whereas miRNAs functioning as oncosuppressor miRs, typically lost or downregulated during cancer, normally assist in the cell cycle arrest [75]. For instance, the miR-17-92 cluster regulates the translation process of E2F transcription factor 1 (E2F1), E2F2, and E2F3, which are key cell proliferation protein regulators; in turn, a negative feedback mechanism regulates the expression of the miR-17-92 cluster. In cancer cells, miR-17-92 overexpression disrupts this negative feedback loop, leading to cell proliferation [76,77]. Conversely, the miR-17-20 cluster, which represses cyclin D1 expression and suppresses breast cancer cell proliferation, has been found to be downregulated in breast tumors [78].

miRNAs are also linked to the core apoptosis pathways in cancer. In fact, there is a growing list of identified miRNAs with both anti-apoptotic and pro-apoptotic properties, which target the central apoptotic genes such as Phosphatase and Tensin Homolog (PTEN), Caspase-9, or B-cell lymphoma 2 (BCL-2). Interestingly, involvement in apoptosis gives miRNAs a major role in cancer drug resistance. For example, miRNA-21 targets PTEN in stomach cancer and breast cancer, promoting cell resistance to a variety of drugs [79,80].

On the other hand, a wide range of miRNAs has been revealed as modulators of the cellular pathways involved in senescence. Senescence is the irreversible state of cellular growth arrest and constitutes a barrier to tumorigenesis since it prevents the malignant proliferation of cells harboring oncogenic DNA mutations [81]. Remarkably, miRNAs commonly associated with senescence have also been involved in human malignancies, such as let-7 miRNAs [82].

In the same way, crucial genes involved in the DNA damage response, which is critical in cancer, are regulated by their specific miRNA. One great example is the linear signaling pathway of N-MYC→miR-421→Ataxia Telangiectasia Mutated (ATM), where the oncogenic transcription factor N-MYC upregulates miR-421, which targets the apical damage sensor kinase ATM. In this fashion, miR-421-mediated ATM downregulation is thought to contribute to N-MYC-induced tumorigenesis in neuroblastoma [83].

Another important cellular process in which miRNAs play an important role is autophagy. Increasing studies have linked miRNAs to autophagic regulation during cancer initiation (such as miR-224 targeting SMAD Family Member 4 (SMAD4) in hepatocellular carcinoma (HCC)) and cancer development (e.g., miR-224-3p targeting RB1-inducible coiled-coil protein (RB1CC1) in cervical tumors) [84]. Autophagy is a multi-step lysosomal degradation process whereby a cell degrades long-lived proteins and damaged organelles. Especially in cancer cells, autophagy serves as a means of temporary survival, a relevant physiological mechanism. However, if cellular stress induces continuous or excessive autophagy, cell death ensues. All in all, miRNAs are involved in several autophagic stages in which they exert a function as oncomiRs or oncosuppressor miRs [85].

### 5.3. Bidirectional Relationship between Epigenetic Alterations and miRNA Dysregulation: Cases with Biological Relevance in Cancer Diseases

As indicated in Section 3, miRNA gene expression is subjected to epigenetic mechanisms, and at the same time, miRNAs have been proved to regulate the expression of epigenetic regulators. As a matter of fact, there is current evidence indicating that dysregulation of miRNAs can lead to aberrant DNA methylation in cancer diseases [86]. Thus, a bidirectional relationship is established between epigenetic alterations and miRNA dysregulation in cancer, often being involved in regulatory loops (Table 1).

#### 5.3.1. miRNAs and Lung Cancer

miRNAs inactivation via promoter DNA methylation has shown biological significance, especially in lung cancer. For instance, aberrant CpG methylation downregulates the expression level of miR-145 in lung adenocarcinoma. miR-145 has been recognized to act as an oncosuppressor miR, having shown to be involved in tumor invasion and progression by targeting C-MYC, Astrocyte Elevated Gene-1 (AEG-1), Epidermal Growth Factor Receptor (EGFR), Nudix Hydrolase 1 (NUDT1), and Octamer-binding transcription factor 4 (OCT4) in LAC [87]. To cite more examples, miR-127 and miR-9 promoter hypermethylation have also been proposed to play a role in non-small cell lung cancer (NSCLC) development and progression [88]. Additionally, miR-34b/c promoter hypermethylation is a frequent event in lung adenocarcinoma, and low levels of miR-34b and miR-34c are associated with distant metastases. Paradoxically, it is important to note that although miR-34b/c downregulation in metastasizing lung adenocarcinomas can be a direct result of increased miR-34b/c promoter hypermethylation, the hypermethylation itself is not associated with metastasizing lung adenocarcinomas [89]. This highlights the complex regulatory networks in which miRNAs play a role in cancer and the difficulty of addressing them. A previous study already stated that miR-34b/c promoter methylation and consequent downregulation is a frequent event in lung adenocarcinomas and that restoration of miR-34b/c expression suppresses cell proliferation, migration, and invasiveness [90]. On the other hand, Brueckner et al. demonstrated that the let-7a-3 promoter could be hypomethylated in human lung cancer, leading to its epigenetic activation and therefore identifying let-7a-3 as a miRNA gene with oncogenic function in lung cancer [91]. However, it is important to note that the vast majority of the literature currently focuses on localized hypermethylation instead of hypomethylation concerning miRNA dysregulation, just as more studies have been conducted on the potential tumor-suppressor genes hypermethylated in cancer rather than on the potential oncogenes hypomethylated in cancer. This is probably because, as we previously described, hypermethylation tends to be in localized gene-associated regions, whereas hypomethylation tends to be generalized to the whole genome, affecting mostly repeated DNA sequences.

With regard to miRNAs contributing to DNA methylation dysregulation, it has been long described in lung cancer that the miR-29 family members target both DNMT3A and DNMT3B. In fact, the relevance of miR-29s was discovered after its reinforced expression in NSCLC cell lines restored the normal patterns of DNA methylation, inducing the re-expression of methylation-silenced tumor-suppressor genes, such as Fragile Histidine Triad Diadenosine Triphosphatase (*FHIT*) and WW Domain Containing Oxidoreductase (*WWOX)*, and inhibiting tumorigenicity in vitro and in vivo [92]. Interestingly, the expression of miR-29a and miR-29b could be partially regulated in a positive feedback loop by DNMT3A and DNMT3B [93].

On the other hand, downregulation of miR-212 correlated to the severity of the disease in lung cancer, and its transcriptional silencing was found to be associated with H3K9me2 and H3K27me3 but not DNA hypermethylation [94]. Furthermore, HDAC5 was found to be aberrantly overexpressed in lung cancer, negatively correlating with miRNA-589 expression. Remarkably, miR-589 was found to target HDAC5 mRNA, regulating important cell cycle and EMT-related genes. Interestingly, it is the hypermethylation of the miR-589 promoter that ultimately leads to the upregulation of HDAC5 [95].

#### 5.3.2. miRNAs and Gastric Cancer

Even though there is less research conducted on gastric cancer (GC) on this topic, it also serves to exemplify the epigenetics–microRNA regulatory networks. With regard to miRNAs activation induced by loss of DNA methylation in GC, Tsai and co-workers demonstrated that abnormal DNA hypomethylation induced overexpression of miR-196b [96]. Later on, it was glimpsed that miR-196b upregulation promoted the proliferation and invasion ability of GC cells by regulating the Phosphatidylinositol 3 kinase (PI3K)/Protein kinase B (AKT)/Mechanistic Target of Rapamycin Kinase (mTOR) pathway [97]. Very recently, miR-196b has also been shown to promote GC progression by targeting Augurin Precursor (ECRG4) [98]. On the other hand, Hashimoto et al. argued that miR-181c could be silenced through methylation in GC, activating its target genes Neurogenic Locus Notch Homolog Protein 2/4 (*NOTCH2/4)* and *KRAS* and therefore contributing to the pathogenesis of GC [99]. Zabaglia et al. also supported that downregulation of miR-181c may play an important role in GC progression by controlling the important genes associated with apoptosis [100]. Remarkably, recent research stated that miR-129-2 was hypermethylated in tumoral tissues of GC patients, suggesting that its methylation was involved in the development of the disease [101]. Hypermethylation of miR-129-2 in primary GC tissues was already reported two decades ago altogether with that of the aforementioned miR-34b. Hypermethylation of miR-129-2 promoter has also been reported in other cancers, such as HCC [102,103], endometrial cancer [104], and ovarian cancer [105].

Interestingly, a novel epigenetic feedback loop between miR-200c and DNMT3A has been described in the carcinogenesis and progression of GC. DNMT3A upregulation is responsible for the hypermethylation of the miR-200c gene promoter in GC, ultimately causing the downregulation of miR-200c. At the same time, miR-200c directly targets DNMT3A and induces endogenous pre-miR-200c and pri-miR-200c re-expression [106].

#### 5.3.3. miRNAs and Ovarian Cancer

In ovarian cancer samples, Chen et al. observed that the increase in the promoter hypermethylation of miR-193a-3p was significantly correlated with the loss of miR-193a-3p expression and tumor stage [107]. Remarkably, by conducting in vivo studies, they concluded that loss of miR-193a-3p could enhance oncogenic capacities via activation of MAPK/ERK signaling, facilitating tumor colonization of metastatic ovarian cancer in peritoneal metastases. Regarding cisplatin resistance in ovarian cancer, Deng and colleagues revealed miR-199a-3p as an upstream regulator of Discoidin Domain Receptor Tyrosine 1 (DDR1) (which confers the malignance and cisplatin resistance of ovarian cancer) that happens to be hypermethylated in ovarian cancer. Thus, the hypermethylated miR-199a-3p gene contributes to tumor aggressiveness and cisplatin resistance through promoting DDR1 expression [108]. Besides, in connection with ovarian cancer metastasis, Vitaly et al. recently showed the involvement of some novel hypermethylated miRNA genes in ovarian metastasis and the inactivation of miR-191 via hypomethylation with a potentially associated oncogenic role [105].

Furthermore, another bidirectional regulation has been described between DNA methyltransferases and miRNAs, with importance in ovarian cancer: a feedback loop between miR-30a/c-5p and DNMT1 that mediates cisplatin resistance [109]. As the authors of this study claim, miR-30a/c-5p is aberrantly methylated and thus silenced by overexpressed DNMT1, which relieves the inhibitory effect of miR-30a/c-5p on DNMT1 and Snail (a key inducer of EMT), leading to cisplatin resistance and partial EMT in ovarian cancer in vitro. On the other hand, re-expression of miR-145 in ovarian cancer cells, which is usually downregulated in this cancer, was shown to inhibit the Warburg effect by targeting DNMT3A and hexokinase-2 (HK2) [110]. Moreover, DNMT3A regulated miR-145 expression through methylation, giving rise to a negative feedback loop. Interestingly, miR-137 mediated the functional link between c-MYC and EZH2, regulating cisplatin resistance in ovarian cancer. The downregulation of miR-137 (which targets *EZH2* mRNA) leads to an increased expression of EZH2, which activates the cellular survival pathways, resulting in resistance to cisplatin [7].

#### 5.3.4. miRNAs and Breast Cancer

Epigenetic-miRNAs regulatory networks have also been described in breast cancer. For instance, the miR-129-2 gene has been observed to be hypermethylated in breast cancer. Furthermore, downregulation of miR-129-2 by promoter hypermethylation has been shown to regulate cell proliferation and apoptosis [111]. Another example can be illustrated by the work of Gacem and co-workers, who determined that miR-124a-1, miR-124a-2, and miR-124a-3 genes were frequently methylated in breast cancer and played a role in tumor growth and aggressiveness [112]. On the other hand, Hu et al. found a hypomethylated miRNA, miR-663, whose overexpression could induce chemoresistance in breast cancer cells [113].

More importantly, almost a decade ago, Xu et al., for the first time, described a negative regulatory circuit between DNMT1 and two miRNAs, miR-148a and miR-152, in breast cancer cells [114]. The downregulation of miR-148a and miR-152 as a consequence of their promoter methylation was inversely correlated with tumor grades and lymph node status in breast cancer tissues. These miRNAs appeared to act as tumor suppressors by targeting Insulin-like Growth Factor 1 Receptor (IGF-1R) and Insulin Receptor Substrate 1 (IRS1), often overexpressed in breast cancer.

In breast cancer, the epigenetic regulation of HOX genes is also remarkable. For instance, the overexpression of the HOXA1 gene is counteracted by the expression of miR-1469, miR-99a, and miR-100 in particular BC contexts. Promoter hypermethylation of HOX genes, such as HOXA5, can also lead to altered expression levels of this gene, causing its silencing [115].

Regarding histone modifications that provoke miRNAs dysregulation, Ryu and co-workers identified miR-708 to be transcriptionally repressed by Polycomb Repressor Complex 2-induced H3K27me3 in metastatic breast cancer. Interestingly, in patients with breast cancer, miR-708 expression was decreased in lymph nodes and distal metastases, suggesting a metastasis-suppressive role [116].

In summary, we have provided several examples of the most frequent epigenetic alterations and miRNA aberrant expression in common cancers and how they are interrelated. Their regulation is dependent on each other. As we have evidenced, the bidirectional regulation between epigenetic mechanisms (especially DNA methylation) and miRNAs has been described in several types of cancer. These epigenetic alterations have been reported at every stage of cancer, from initiation to progression, in metastasis, and in resistance to oncologic therapies. In addition, it has been proved to affect the course of these events profoundly. This highlights their importance in cancer, and thus, the need to take them into account when trying to improve our knowledge of tumoral malignancies.

## 6. Clinical Applications: miRNAs Epigenetics in Cancer

Biomarkers are indicators of either physiological or pathological biological processes. An acceptable biomarker should be accurate and highly reproducible in standardized cost-effective assays. Besides, it should be preferably measured from minimally invasive samples and provide valuable information for the patient’s clinical management. Many miRNAs have been found to be aberrantly expressed in different malignancies. As we have shown, epigenetic mechanisms, such as hypo/hypermethylation of promoter CpG islands or histone post-transcriptional modifications, regulate miRNA expression. The detection of these deregulated mechanisms may serve as promising diagnostic and prognostic biomarkers in cancer as well as novel therapeutic strategies.

### 6.1. miRNAs Methylation as Diagnostic Biomarkers

Many studies have highlighted the potential benefits of implementing methods to evaluate aberrant miRNA promoter methylation patterns in biological samples as a strategy for early detection of cancer. However, few studies describing novel diagnostic biomarkers based on miRNAs methylation are reported in the literature to date. This section summarizes those that we considered most promising in the context of cancer diagnosis (Table 2).

Toiyama and co-workers evaluated the potential of miR-1, miR-9, miR-124, miR-137, and miR-34b/c methylation levels as diagnostic biomarkers in ulcerative colitis (UC)-associated colorectal cancer. Methylation of the aforementioned miRNAs was increased in cancer tissues and dysplasia compared to UC non-neoplastic tissues. The combination of all miRNAs allowed for more robust discrimination of colorectal carcinoma patients. More importantly, they found that this signature could accurately identify patients with ulcerative colitis at risk of developing colorectal carcinoma (CRC), with high sensitivity and specificity [118].

DNA methylation-based silencing of miR-124 was shown to be a marker for improved detection of cervical cancer and its high-grade precursor lesions [119]. Subsequently, several studies have validated the use of a methylation-based signature composed of a combination of miR-124 and other genes (MAL/miR-124-2, FAM19A4/miR-124-2...) as a triage test for the identification of premalignant lesions (cervical intraepithelial neoplasia) in high-risk human papillomavirus-positive women [120,121]. Furthermore, FAM19A4/miR-124-2 methylation analysis in large cohorts of patients confirmed its value as a high-sensitivity screening method for the diagnosis of cervical cancer [122,123].

To date, many efforts have been made to identify alterations in the methylation patterns in tissue samples from cancer patients. However, given the costs and risks associated with surgical biopsy, identifying these biomarkers in liquid biopsy provides great benefit for the patients. In addition, biological fluids, such as plasma, serum, urine, saliva, or stool, have been shown to provide valuable information for the diagnosis of a wide range of tumors.

Using urine sediments, methylation of miRNAs has also been demonstrated to be useful to diagnose different genitourinary carcinomas. For instance, miR-193b promoter methylation levels allow the detection of prostate cancer with 91.6% sensitivity and 95.7% specificity, providing an overall accuracy of 92.9% [124]. Comparing the methylation levels of miR-30a-5p in urine from patients with renal clear carcinoma (RCC) and asymptomatic controls, Outeiro-Pinho and colleagues have also shown the potential utility of this biomarker in the diagnosis of RCC. The overall accuracy of this assay was not very high (67%), but the results were validated in the second cohort of 171 RCCs [125]. Moreover, a panel of two microRNA methylated promoters composed of miR-663a and miR-129-2 was shown to accurately detect urothelial carcinomas in urine (85.85% accuracy) [126].

Lu and co-workers have also demonstrated that methylation levels of miR-129-2 were increased in HCC compared to adjacent normal tissue. Moreover, miR-129-2 methylation was detected in plasma from HCC patients but not in plasma from liver cirrhosis patients or healthy individuals, which implies a potential utility of this biomarker as an early diagnostic marker for HCC [102]. Furthermore, miR-17-5p methylation level allows the discrimination of patients with pancreatic tumors from healthy controls with extremely high specificity and sensitivity [127].

Additionally, serum-circulating DNA was used to demonstrate the value of miR-34b/c methylation for the diagnosis of malignant pleural mesothelioma with high specificity and moderate sensitivity [128,129]. Abnormal methylation of CpG islands of miR-34b/c promoter has been proposed as a potential biomarker for detecting CRC using fecal samples [130,131]. Kalimutho and co-workers found that 75% of fecal specimens from CRC patients were positive for promoter methylation of miR-34b/c, whereas only 16% of patients with high-grade dysplasia and 13% of healthy individuals showed this alteration [130]. Similarly, Wu et al. compared the positive rate of miR-34b/c methylation in fecal samples from CRC patients and healthy individuals and showed that the sensitivity and specificity for screening CRC were very high (95% and 100%, respectively) [131].

Detection of miRNA gene promoter hypermethylation in oral rinses has also been investigated. Promoter methylation of miR-137 is a relatively common event in head and neck squamous cell carcinoma (HNSCC), and its presence in oral rinses from these patients has been demonstrated. Moreover, HNSCC patients had nearly five times the odds of having miR-137 promoter methylation compared to the normal oral mucosa of control subjects. Thus, the miR-137 promoter methylation level in oral rinses distinguished HNSCC patients from healthy individuals with high specificity but low sensitivity [132].

### 6.2. Epigenetic Regulation of miRNAs as Prognostic Biomarkers

Discrimination of cancer patients with an aggressive biology could assist the clinicians in the management of these patients. As a result, a high number of prognostic markers have been identified, but unfortunately, trials that validate and confirm the utility of these markers are still lacking in most cases. Thus, the identification of novel tools that allow a more accurate prognostication of the patient is needed.

In 2008, Lujambio et al. showed that methylation of miRNAs was correlated with the metastatic behavior of tumors in different organs. Lujambio et al. demonstrated that hypermethylation of the miR-34b/c, miR-148a, and miR-9-3 CpG islands was significantly associated with the presence of lymph node metastasis in melanoma, lung, and breast cancer [133]. Subsequent studies confirmed [134,135] and extended these results to other miRNAs and malignancies. Specifically, the correlation between lymph node metastasis and aberrant methylation was also observed in CRC for miR-9-1 [67] and miR-34a [136] and in invasive breast ductal carcinomas for miR-124a [112].

Given that lymph node metastasis is often associated with tumor recurrence and poor survival, the prognostic value of a plethora of miRNAs methylation has been evaluated (Table 3). In this context, hypermethylation of miR-124 and miR-9 was shown to be associated with an increased risk of recurrence in clear cell RCC [137,138]. In contrast, in breast cancer, miR-124 methylation levels were associated with different survival rates according to the age of the patients. The study concluded that miR-124 hypomethylation was a poor prognostic marker in young breast cancer patients (≤35 years old) as opposed to the longer survival rates found in older patients (>50 years old) [139].

In tumors of the respiratory tract, the prognostic value of miR-34b/c methylation is one of the most frequently investigated. In 2011, Wang et al. showed that aberrant miR-34b/c DNA methylation was an independent prognostic marker of stage I NSCLC. In this study, the association between altered DNA methylation of miR-34b/c and shorter recurrence-free and overall survival was demonstrated in a large series of 161 patients. This proved to be very useful for selecting a subset of stage I tumors with poor outcomes, which could benefit from additional therapy after resection [140]. Subsequently, these results were confirmed in 140 lung adenocarcinoma patients. They evaluated the prognostic value of miR-34b/c methylation in an exploratory set of 58 LAC lung adenocarcinomas and validated their results in a confirmatory cohort of 82 patients. Moreover, miR-34b/c methylation was also a prognostic marker for stage I lung adenocarcinoma patients [90]. Besides, Kim et al. confirmed the prognostic value of miR-34b/c in NSCLC and demonstrated a combined effect of miR-34b/c and miR-124-3 methylation patterns for the prognosis of NSCLC. Overall survival decreased as the number of methylated miRNAs increased; i.e., patients with two methylations exhibited significantly poorer overall survival than patients with none or one methylation [141]. In line with these results, a profile composed of the methylation of five genes (miR-152, miR-9-3, miR-124-1, miR-124-2, and miR-124-3) was analyzed in NSCLC. Longer progression-free survival was proved in patients with none or one methylation compared to two or more [142]. The association between survival and hypermethylation of other miRNAs, such as miR-127 or miR-145, has also been shown in lung cancer patients [87,88]. In addition, the hypermethylation of miR-137 was associated with a shorter overall survival in HNSCC [143].

In CRC, hypermethylation of miR-34a promoter CpG islands was also strongly associated with metastasis to the liver [136]. Moreover, the expression of miR-148a was inversely correlated with its promoter methylation status. Both markers were pointed out as independent predictors of survival in adjuvant-treated stage IV CRC patients [144].

The prognostic value of the methylation status of different miRNAs has also been investigated in hematological malignancies. Specifically, epigenetic silencing of miR-124a has been found to correlate with a higher recurrence rate and mortality rate in Acute Lymphoblastic Leukaemia (ALL), being an independent predictor for disease-free survival and overall survival [145]. Moreover, the same group analyzed the hypermethylation profile of 11 CpG islands associated with several miRNAs (miR-124a1, miR-124a2, miR-124a3, miR-34b/c, miR-9-1, miR-9-3, miR-10b, miR-203, miR-196b, miR-9-2, and miR-132/212) in the same cohort of ALL patients and they found statistically significant differences in outcome between non-methylated and methylated ALL patients (methylation in at least one miRNA) [146]. Other groups have also examined the prognostic role of various miRNAs methylation in different lymphoid malignancies and showed that miR-129-2 and miR-340 methylation adversely impacted the survival factors in chronic lymphocytic leukemia multiple myeloma, respectively [147,148]. Moreover, aberrant miR-137 methylation was shown to be associated with shorter progression-free survival in myeloma [149].

### 6.3. Epigenetic Regulation of miRNAs as a Therapeutic Strategy in Cancer

Modulation of miRNAs expression has emerged as a promising strategy for cancer management. With the aim of restoring the expression levels of oncosuppressor miRs in cancer cells, different epigenetic drugs have been tested on different tumor models, and their tumor-suppressive effects have been evaluated [133,135,150]. Some of these drugs, such as 5-Aza derivatives (Azacitidine and 5-Aza-dC) or HDAC inhibitors (Vorinostat, Panobinostat, Belinostat, and Romidepsin), have already been approved by the U.S. Food and Drug Administration for the treatment of different hematologic malignancies. Nowadays, an extension of their usage to solid tumors is being pursued by different pharmaceutical companies. The discovery of novel epigenetic drugs is also receiving great attention. A wide range of epigenetic-based drugs are being tested in preclinical and clinical trials (for a review, see [151]). These drugs trigger a global effect in coding and non-coding genes, and thus their impact is difficult to be attributed to specific genes. Few reports have linked specific responses to epigenetic drugs and miRNAs expression in treated patients. Recently, Berg et al. found that the hypomethylating agents azacitidine and decitabine significantly upregulated the expression of miR-125a associated with anti-leukemic effects. These data were validated in chronic myelomonocytic leukemia patients, where higher levels of miR-125a were observed after treatment with the hypomethylating agents. Importantly, the increase was particularly pronounced in the responders to these drugs [152]. More insight into the link between the mechanism (mode of action) of the epigenetic drugs and the miRNAs in cancer will provide new opportunities in the development of new strategies for cancer therapy.

## 7. Conclusions

This review highlighted the functional implications of the epigenetic alterations and miRNA dysregulation in cancer diseases. Given the implications of miRNA in cancer-related pathways and their described oncogenic or tumor-suppressive roles, their dysregulation seems crucial to fully understand neoplasia. The existing bidirectional regulation between DNA methylation, histone modifications, and miRNAs places epigenetics as one of the central pillars of carcinogenesis. Deciphering the epigenetic regulation of mRNAs in cancer diseases provides more insight into tumor initiation and progression and gives rise to a wide range of potential clinical applications. As we have overviewed, this is especially reflected by the large number of miRNA genes with aberrant methylation that have been proposed as putative biomarkers for diagnosis or prognosis in cancer diseases.

## Figures and Tables

**Table 1:** Epigenetic bidirectional regulation between epigenetic mechanisms and miRNAs disrupted in some types of cancer diseases, with biological consequences.

Type of Tumor	miRNAs Inactivation via DNA Hypermethylation	miRNAs Activation via DNA Hypomethylation	DNA Methylation Induced by miRNAs and Feedback Loop	miRNAs Dysregulation via Histone Modifications and Vice Versa	Targets/Pathways Affected by miRNAs Dysregulation with Potential Clinical Implications
Lung cancer	miR-145 [87]; miR-127, miR-9 [88]; miR-34b/c [89,90]	miR let-7a-3 [91]	miR-29 ⇒DNMT3A&DNMT3B [92]; miR-29a/b ⇔ DNMT3A & DNMT3B [93]	H3K9me2 & H3K27me3 ⇒ miR-212 [94]; miR 589 ⇒ HDAC5 [95]	miR-145 ⇒ c-Myc, AEG-1, EGFR, NUDT1 [87]
Gastric cancer	miR-181c [99] miR-129-2 [101,117]	miR-196b [96]	miR-200c ⇔ DNMT3A [106]	HDAC ⇒ miR-127 [33]	miR-196b ⇒PI3K/ AKT)/ mTOR pathway [97]; miR-196b ⇒ ECRG4 [98]
Ovarian cancer	mir-193a-3p [107], miR-199a-3p [108]	miR-191 [105]	miR-30a/c-5p ⇔ DNMT1 [109]	miR-145 ⇒ DNMT3A [110]; miR-137⇒ EZH2 [7]; miR-101-3p, let-7e-5p, miR-26a-5p, miR-98-5p, miR-141-3p ⇔ EZH2 [43]	miR-193a-3p ⇒ MAPK/ERK [107]; miR-199a-3p ⇒ DDR1 [108]; miR-30a/c-5p ⇒ SNAIL [109]; miR-145 ⇒ DNMT3A, HK2 [110]
Breast cancer	miR-129-2 [111]; miR-124a-1, miR-124a-2 & miR-124a-3 [112]	miR-663 [113]	miR-148a & miR-152 ⇔ DNMT1 [114]	H3K27me3 ⇒ miR-708 [116]	miR-129-2 ⇒ BCL2L2 [111]; miR-148a & miR-152 ⇒ IGF-1R, IRS1 [114]

**Table 2:** miRNAs methylation as a biomarker for cancer diagnosis.

Cancer Type	Type of Marker	miRNA	Source of miRNA	Sensibility	Specificity	Reference
CRC	Single	miR-1	Tissue	84.6%	75.8%	[118]
		miR-9		61.5%	77.4%	
		miR-124		76.9%	67.7%	
		miR-137		76.9%	80.6%	
		miR-34b/c		100%	56.5%	
Prostate carcinoma	Single	miR-193b	Urine	91.6%	95.7%	[124]
RCC	Single	miR30a-5p	Urine	83%	53%	[125]
Urothelial carcinomas	Signature	miR-663a	Urine	87.7%	84%	[126]
		miR-129-2				
Pancreatic carcinoma	Single	miR-17-5p	Plasma	ND	ND	[127]
Pleural mesothelioma	Single	miR34b/c	Serum	67%	77%	[128,129]
				65.7%	94.9%	
CRC	Single	miR-34b/c	Feces	ND	ND	[130,131]
				95%	100%	
CRC	Single	miR-34a	Feces	76.8%	93.6%	[130,131]
HNSCC	Single	miR-137	Oral rinses	46.5%	81.1%	[132]

CRC: Colorectal carcinoma; RCC: Renal carcinoma; HCC: Hepatocellular carcinoma; HNSCC: Head and Neck squamous cell carcinoma; ND: Not determined.

**Table 3:** miRNAs methylation as a biomarker for cancer prognosis.

Cancer Type	Type of Marker	miRNA	End Point	Univariate Analysis	Adjusted Analysis	Reference
ccRCC	Single	miR-9-1	RFS	*p* = 0.034	HR = 2.74 95% CI = 0.78–9.60	[137,138]
	Single	miR9-3	RFS	*p* = 0.007	HR = 5.85 95% CI = 1.30–26.35	
ccRCC	Single	miR-124-3	RFS	*p* = 0.0005	NA	[137,138]
Breast cancer	Single	miR-124-2	OS	*p* = 0.0009	HR = 3.23 *p* = 0.001	[139]
NSCLC	Single	miR34b/c	RFS	*p* = 0.017	HR = 2.60 95% CI = 1.34–5.06 *p* = 0.005	[140]
			OS	*p* = 0.010	HR = 2.20 95% CI = 1.03–4.67 *p* = 0.027	
NSCLC	Single	miR34b/c	RFS	*p* = 0.0003	HR = 2.16 95% CI = 1.32–3.52 *p* = 0.002	[90]
			OS	*p* = 0.016	HR = 1.79 95% CI = 1.07–3.02 *p* = 0.027	
NSCLC	Signature	miR-34b/c	OS	*p* < 0.0001	HR = 4.44 95% CI = 2.15–9.18 *p* < 0.0001	[141]
		miR-124-3				
NSCLC	Signature	miR-152	RFS	*p* = 0.0177	NA	[142]
		miR-9-3				
		miR-124-1				
		miR-124-2				
		miR-124-3				
NSCLC	Single	miR-127	OS	*p* = 0.010	HR = 1.97 95% CI = 1.15–3.40 *p* = 0.014	[88]
HNSCC	Single	miR-137	OS	*p* = 0.046	HR = 3.68 95% CI = 1.01–13.38 *p* < 0.05	[143]
CRC	Single	miR-148a	RFS	*p* = 0.020	NA HR = 3.046 95% CI = 1.56–5.93 *p* = 0.0011	[144]
			OS	*p* = 0.0015		
ALL	Single	miR-124a	RFS	*p* = 0.001	*p* < 0.001	[145]
			OS	*p* < 0.001	*p =* 0.005	
ALL	Signature	miR-124a1 miR-124a2 miR-124a3 miR-34b/c miR-9-1 miR-9-2 miR-9-3 miR-10b miR-203 miR-196b miR-132/212	RFS OS	*p* < 0.001 *p* < 0.001	*p* < 0.001 *p* < 0.001	[146]
Chronic lymphocytic leukemia	Single	miR-129-2	OS	*p* = 0.004	NA	[147]
Multiple myeloma	Single	miR-340	OS	*p* < 0.001	HR = 8.983 95% CI = 2.2–36.63 *p* = 0.002	[148]
Multiple myeloma	Single	miR-137	RFS	*p* = 0.043	NA	[149]

CRC: Colorectal carcinoma; HCC: Hepatocellular carcinoma; LAC: Lung adenocarcinoma; NSCLC: Non-small cell lung cancer; ccRCC: Clear cells renal carcinoma; HNSCC: Head and neck squamous cell carcinoma; ALL: Acute lymphoblastic leukemia; RFS: Recurrence-free survival; OS: Overall survival; HR: Hazard ratio.
